# Risk of selection bias assessment in the NINDS rt-PA stroke study

**DOI:** 10.1186/s12874-022-01651-4

**Published:** 2022-06-15

**Authors:** Ravi Garg, Steffen Mickenautsch

**Affiliations:** 1 Department of Neurology, Division of Neurocritical Care, Loyola University Chicago Stritch School of Medicine, 2160 S First Avenue, Maywood, IL 60153 USA; 2 Faculty of Dentistry, University of the Western Cape, Francie van Zijl Avenue, Tygerberg, Cape Town, 7505 South Africa; 3 Honorary/Department of Community Dentistry, School of Oral Health Sciences, Faculty of Health Sciences, University of the Witwatersrand, 7 York Rd., Parktown, Johannesburg, 2193 South Africa; 4 Review Center For Health Science Research, 84 Concorde Road East, Bedfordview, Johannesburg, 2008 South Africa

**Keywords:** Acute ischemic stroke, Selection bias, Alteplase

## Abstract

**Objectives:**

The NINDS rt-PA Stroke Study is frequently cited in support of alteplase for acute ischemic stroke within 3 h of symptom onset. Multiple post-hoc reanalyses of this trial have been published to adjust for a baseline imbalance in stroke severity. We performed a risk of selection bias assessment and reanalyzed trial data to determine if the etiology of this baseline imbalance was more likely due to random chance or randomization errors.

**Methods:**

A risk of selection bias assessment was conducted using signaling questions from the Cochrane Risk of Bias 2 (ROB 2) tool. Four sensitivity analyses were conducted on the trial data based on the randomization process: assessment of imbalances in allocation in unique strata; adherence to a pre-specified restriction on randomization between time strata at each randomization center; assessment of differences in baseline computed tomography (CT) results in unique strata; and comparison of baseline characteristics between allocation groups within each time strata. A multivariable logistic regression model was used to compare reported treatment effects with revised treatment effects after adjustment of baseline imbalances identified in the sensitivity analyses.

**Results:**

Based on criteria from the ROB 2 tool, the risk of bias arising from the randomization process was high. Sensitivity analyses found 11 of 16 unique strata deviated from the expected 1:1 allocation ratio. Three randomization centers violated an apriori rule regarding a maximum difference in allocation between the time strata. Three unique strata had imbalances in baseline CT results that prognostically favored alteplase. Four imbalances in baseline characteristics were identified in the 91–180-min time stratum that all prognostically favored alteplase and were consistent with a larger alteplase treatment effect size compared to the 0–90-min time stratum. After adjustments for baseline imbalances, all reported treatment effects were reduced. Three out of seven originally positive reported results were revised to non-significant.

**Conclusion:**

This risk of selection bias assessment revealed a high risk of selection bias in the NINDS rt-PA Stroke Study. Sensitivity analyses conducted based on the randomization process supported this assessment. Baseline imbalances in the trial were more likely due to randomization errors than random chance. Adjusted analyses accounting for baseline imbalances revealed a reduction in reported treatment effects supporting the presence of selection bias in the trial. Treatment decisions and guideline recommendations based on the original treatment effect reported in the NINDS rt-PA Stroke Study should be done cautiously.

## Background

The NINDS rt-PA Stroke Study is a phase 3 randomized controlled trial (RCT) that is frequently cited in support of alteplase for acute ischemic stroke within 3 h of symptom onset [1, 2]. This study was published as a two-part RCT with distinct primary endpoints and different analytical methods for each part [2]. The trial employed permuted block randomization with various block sizes and 1:1 allocation, stratifying by two covariates: time of symptom onset (0-90 min or 91-180 min) and clinical center for a total of 16 unique strata [2]. A coordinating center was responsible for generating randomization schedules [3]. Each of the 8 clinical centers shared the randomization schedules with each of their associated treatment centers (hospitals) where participants were randomized [3]. Clinical centers could also serve as treatment centers where participants were randomized; and some exclusively served as their own treatment center [3]. The single coordinating center served as both its own clinical center and single treatment center [3]. When a participant was randomized at a treatment center, the associated clinical center was contacted and responsible for ensuring that all other treatment centers associated with that clinical center used the next allocation from the shared randomization schedule [3]. Randomization was additionally restricted by center such that the number of participants randomized in one time stratum (e.g., 91-180 min) could not be greater than the number of  participants randomized in the other time stratum (e.g., 0-90 min) by 3 or more participants [3]. This restriction was placed in anticipation that a greater number of participants would be eligible in the 91–180-min stratum [3]. If this occurred, otherwise eligible participants would be excluded until the difference in the number of participants enrolled in the time strata was < 3.

Allocation concealment was maintained by using manila envelopes attached to pre-packed boxes of study drug [3]. The box had an attached participant ID that was to be matched with the randomization schedule [3]. For alteplase, both the vial contents and instructions regarding reconstitution were present in the study protocol. The study protocol and the product licensing application (PLA) note “matched placebo” was used, but vial contents were not listed [3]. When an eligible participant was identified the investigators would notify the pharmacy of possible enrollment and the appropriate pre-pack was identified based on the randomization schedule [3]. The reconstituted study drug was then brought to the location of the participant where the investigator would complete screening, including evaluation of a baseline computed tomography (CT) scan, and determine eligibility [3]. One CT scan criterion, presence of intracerebral hemorrhage, was considered an absolute contraindication. If an investigator subjectively felt that the baseline CT scan results were inconsistent with the self-reported time of symptom onset, they were allowed to re-question the participant and/or family on this eligibility criteria [3]. A participant was considered randomized only when the study drug started infusing [3]. Because the study drug was sometimes prepared for participants that were ultimately not randomized, investigators reused the same participant ID for multiple different study pre-packs, presumably of the same contents [3]. An additional sequence ID was attached to each pre-pack such that if a pre-pack was reconstituted but unused, a pre-pack with the same participant ID and different sequence ID was available for use. This would theoretically ensure that if a vial was wasted on an ineligible participant there would be no skipped allocations.

In total, 22 participants had allocations changed due to randomization errors such as randomization out-of-order and randomization from the wrong stratum [3]. 16 total envelopes were unblinded [3]. 12 alteplase allocated participant envelopes were unblinded compared to 4 placebo allocated participant envelopes [3].

A total of 16,009 participants were screened of which 624 were randomized (3.9%). The most common exclusion reason was “time from onset too long” [3]. In each time of symptom onset stratum, an allocation imbalance of 12 participants was present [2]. In the 0–90-min stratum, 157 participants were allocated alteplase compared to 145 placebo participants [2]. And, in the 91–180-min stratum, 167 participants were allocated placebo compared to 155 alteplase participants [2].

Following the original publication, multiple post-hoc reanalyses were published due to a baseline imbalance in the National Institute of Health Stroke Scale (NIHSS) score which is the most important prognostic factor following stroke [4, 5]. These post-hoc reanalyses have assumed that this baseline imbalance was due to random chance and not randomization errors. A detailed risk of selection bias assessment to judge this assumption, however, has never been published. The purpose of this study is to assess the risk of selection bias in the NINDS rt-PA Stroke Study.

## Methods

The NINDS rt-PA Stroke Study publication; study protocol; United States Food and Drug Administration (FDA) product licensing application (PLA); and participant level data were reviewed for this risk of bias assessment [2, 3]. The applied risk assessment included a qualitative risk of bias appraisal, a quantitative sensitivity analyses based on the randomization process, and an adjusted analysis to compare reported alteplase treatment effects to revised effects.

### Risk of selection bias appraisal

The risk of selection bias was appraised using signaling questions from the Cochrane Risk of Bias 2 (RoB 2) tool that address systematic error arising from the randomization process [6].

### Sensitivity analyses

Four sensitivity analyses were performed based on the randomization process using participant level data:

(i) Assessment of imbalances in allocation in unique stratum for which the expected ratio was 1:1
(ii) Adherence to a pre-specified restriction on randomization between time strata such that the number of participants randomized in one time stratum (e.g., 91-180 min) could not be greater than the number of participants randomized in the other time stratum (e.g., 0-90 min) by 3 or more participants at each clinical center
(iii) Assessment of differences in baseline CT results in unique stratum that may suggest deterministic allocation because the protocol allowed investigators to exclude participants based on their subjective interpretation of the baseline CT scan
(iv) Comparison of baseline characteristics between allocation groups within each time strata for which the trial publication reported different effect sizes

Continuous parametric variables were compared using a t-test while a chi-square test with continuity correction was used to compare categorical variables. A *p* value ≤.05 was considered significant for hypothesis testing.

### Adjusted analysis for revised alteplase treatment effects

To assess the potential effect of baseline imbalances on reported alteplase treatment effects an adjusted analysis was performed using multivariable logistic regression. Baseline imbalances determined in sensitivity analyses were chosen as independent variables in addition to the treatment allocation variable. A separate analysis was performed for each of the four dichotomous outcomes in the time strata as originally reported: a modified Rankin scale (mRS) score of 0-1; a Barthel index (BI) of 95 or 100; an NIHSS ≤1; and a Glasgow Outcome Scale (GOS) of 1.

The publicly available NINDS rt-PA Stroke Study data was used for all analyses for which a detailed data description has been published [7]. The data includes 65 categorical and 36 numeric variables.

## Results

### Risk of selection bias appraisal

The response to the signaling questions regarding random allocation sequence, allocation concealment, and baseline differences were answered probably yes; probably no; and yes respectively (Table 1). Based on the criteria from the ROB 2 tool the risk of bias arising from the randomization process was high.

**Table 1:** Risk of bias arising from the randomization process

Signaling Question	Response	Bias	Justification from Trial Publication or Product Licensing Application	Remarks
Was the allocation sequence random?	Probably Yes	Lower risk of Bias	“A permuted-block design with blocks of various sizes was used for randomization, with patients stratified according to clinical center and time from the onset of stroke to the start of treatment (0-90 or 91-180 min)” [2].	No information on method used for random sequence generation.
Was the allocation sequence concealed until participants were enrolled and assigned to interventions?	Probably No	Higher Risk of Bias	“The randomization process was decentralized” [3].	Randomization was done at treatment centers (*N* = 39) but required coordination by clinical centers (*N* = 8) and a coordinating center.
			“There are 16 patients of the total 624 reported as having been unblinded during the study. This includes 12 patients in the Activase group, and 4 in the placebo group” [3].	Reasons for unblinding envelopes available for 8/16 participants.
			“Blinding was incorporated into the studies by using blind labeled vials and identical administration regiments for the treatment arms” [3].	Contents of matched placebo used to generate foaming reaction unreported.
Did baseline differences between intervention groups suggest a problem with the randomization process?	Yes	Higher Risk of Bias	N/A	4 Baseline imbalances identified between groups prognostically favoring alteplase in the 91-180 min stratum were consistent with a larger treatment effect compared to the 0-90 min stratum.

### Sensitivity analyses

The results of the sensitivity analyses were as follows:

(i) In total 11/16 unique stratum deviated from the expected 1:1 allocation in treatment groups
(ii) Three out of eight centers had a ≥ 3 participant difference between the time strata violating a pre-specified rule regarding allocation between time strata at each randomization center
(iii) Of 16 unique stratum, 3 strata had imbalances in baseline CT results (Table 2). All four imbalances in baseline CT results prognostically favored alteplase
(iv) Four imbalances in baseline characteristics were identified in the 91–180-min stratum; and none were identified in the 0–90-min stratum (Table 3). Alteplase allocated participants were more likely to be receiving aspirin prior to treatment (*p* = .002); less likely to have a hyperintense artery sign (*p* = .004) on baseline CT; had a lower mean NIHSS score (*p* = .021); and were more likely to have a small vessel ischemic stroke subtype (*p* = .045). These baseline differences that all prognostically favored alteplase were consistent with the reported stratified effects in the study publication for which a larger alteplase treatment effect is noted in the 91–180-min stratum [mRS < 2, Odds Ratio: 2.4 (1.5-3.7)] compared to the 0–90-min stratum [mRS < 2, Odds Ratio: 1.7 (1.0 – 2.6)].

**Table 2:** Imbalances in baseline computed tomography results

Center ID	Time Stratum	Baseline Differences (% Placebo vs. % Alteplase) or (Placebo Mean vs. Alteplase Mean)	Allocation Difference (Δ)
3	0-90 Min	Loss of Grey White Differentiation (35% vs. 0%, *p* = .020)	Alteplase > Placebo (1)
4	0-90 Min	Abnormal Baseline CT (62% vs. 32%, *p* = .020)	Alteplase > Placebo (5)
4	91-180 Min	Hyperintense Artery Sign (24% vs. 3%, *p* = .025)	Placebo > Alteplase (3)
		Old Lesion Volume (3.1 ± 7.2 vs. .35 ± 1.6, *p* = .025)

**Table 3:** Baseline imbalances in time strata

Time Stratum	Baseline Differences (% Placebo vs. % Alteplase) or (Placebo Mean vs. Alteplase Mean)	Allocation Imbalance (Δ)	Effect Size for a Modified Rankin Scale Score of 0-1
0-90 Min (*N* = 302)	None	Alteplase > Placebo (12)	Risk Ratio: 1.4 (1.0 – 1.9)
			Odds Ratio: 1.7 (1.0-2.6)
91-180 Min (*N* = 322)	Aspirin (24% vs. 41%, *p* = .002)	Placebo > Alteplase (12)	Risk Ratio: 1.8 (1.3-2.4)
	Hyperintense Artery Sign (20% vs. 8%, *p* = .004)		
	NIHSS Baseline (15.4 ± 6.9 vs. 13.5 ± 7.6, *p* = .021)		Odd’s Ratio: 2.4 (1.5 – 3.7)
	Small Vessel Ischemic Stroke (11% vs. 19%, *p* = .045)		

### Adjusted analysis for revised alteplase treatment effects

In the 0–90-min stratum, loss of grey-white differentiation, abnormal baseline CT, and treatment allocation were chosen as independent variables for multivariable logistic regression to determine adjusted odds ratios (aOR) and 95% confidence intervals (CI) for revised treatment effects (Table 4). Compared to the originally reported positive treatment effects, the revised treatment effect for the mRS score was no longer significant (*p* = .059). There was a small non-significant treatment effect reduction in the BI (*p* = .020), GOS (*p* = .097), and NIHSS score (*p* = .018) endpoints. Since there was ambiguity in the trial’s data dictionary regarding whether the definition of the abnormal baseline CT variable already included participants with loss of grey-white differentiation, the analysis was repeated without the loss of grey-white differentiation variable; and revealed identical results.

**Table 4:** Originally reported effect sizes compared to revised effect sizes derived from the adjusted analysis

Time Stratum	Endpoint	Reported Effect Size	Revised Effect Size
0-90 Min (*N* = 302)	Barthel Index	OR 1.8 (1.2-2.9), *p* = .010	aOR 1.7 (1.1 – 2.8), *p* = .020
	Modified Rankin Scale	OR 1.7 (1.0-2.6), *p* = .035	aOR 1.6 (1.0-2.6), *p* = .059
	Glasgow Outcome Scale	OR 1.6 (1.0-2.5), *p* = .057	aOR 1.5 (.9- 2.4), *p* = .097
	NIHSS	OR 2.0 (1.2-3.4), *p* = .008	aOR 1.9 (1.1 – 3.3), *p* = .018
91-180 Min (*N* = 322)	Barthel Index	OR 1.6 (1.1-2.5), *p* = .026	aOR 1.5 (.9-2.5), *p* = .136
	Modified Rankin Scale	OR 2.4 (1.5-3.7), *p* < .001	aOR 2.3 (1.3 – 4.0), *p* = .003
	Glasgow Outcome Scale	OR 2.0 (1.3-3.2), *p* = .002	aOR 1.9 (1.1 – 3.2), *p* = .019
	NIHSS	OR 2.0 (1.2-3.2), *p* = .008	aOR 1.6 (.9 – 2.8), *p* = .114

In the 91–180-min stratum, aspirin prior to treatment; presence of a hyperintense artery sign; baseline NIHSS score; small vessel ischemic stroke subtype; old lesion volume; and treatment allocation were chosen as independent variables for multivariable logistic regression to determine aOR and 95% CI for revised treatment effects (Table 4). Compared to the originally reported positive treatment effects, the revised treatment effects for the BI (*p* = .136) and NIHSS score (*p* = .114) were no longer significant. There was a small non-significant treatment effect reduction in the mRS (*p* = .003) and GOS (*p* = .019) endpoints. In total, 4 out of 8 originally reported treatment effects were non-significant including 3 out of 7 that were originally reported positive and revised to non-significant after covariate adjustment.

## Discussion

Unbiased randomization is essential to ensure the internal validity of an RCT. Selection bias due to flawed randomization is a serious threat to internal validity that is difficult to correct by statistical analysis without a corresponding downgrade in the level of evidence [8]. Multiple facets of the NINDS rt-PA Stroke Study suggest the study suffered from baseline imbalances due to randomization errors and not random chance. These facets that support a high risk of selection bias may be understood as trial design elements and inadequate reported information a priori; and randomization errors and baseline imbalances a posteriori.

The trial employed stratified block randomization using varying blocks sizes. The potential for selection bias using this randomization process has been thoroughly described [9–11]. Contrary to popular belief, varying the block sizes does not sufficiently guard against non-random allocation [9, 11–13]. Key unreported information included how the random sequence was generated and contents of matched placebo. The study protocol provided no details of how allocation concealment was maintained at the vial level. As noted in the manual of procedures, alteplase characteristically foams upon reconstitution. To maintain allocation concealment, it would be essential to ensure placebo matched this foaming reaction. Complications unique to alteplase administration such as gum bleeding or angioedema may have also compromised allocation concealment.

Results from the actual randomization process favor randomization errors over chance imbalances. Allocation concealment failed for 16 participants in which envelopes were prematurely opened. Only half were opened for safety concerns while the other half had no documented reasons for unblinding. The resultant effect of unblinding on random allocation is similar. Compared to trials in which allocation concealment was adequate, trials in which allocation concealment was compromised have larger treatment effect estimates [14]. Additionally, the cross-over ratio due to randomization errors was highly unilateral. Of the 22 participants that had allocations changed, 21 of these involved a participant that should have received alteplase, but instead received placebo. If, for example, the chance of a cross-over under a permuted block design is assumed to .5, the probability of this cross-over due to chance is very low (*p* = .0000052).

Our sensitivity analyses further support baseline imbalances occurred due to randomization errors over chance. Despite using stratified block randomization, for which the primary purpose is to force an equal number of participants in each allocation group within strata, there were imbalanced allocations in 11/16 unique strata. Allocations appear similar in the baseline characteristics table of the original publication due to subdivision into study parts. The addition of a second study part, however, was decided the middle of a continuous enrollment process for analytical purposes. Randomization was uninterrupted and shared between the parts; and therefore, the separation of the study into parts does not reflect the allocation or covariate balance [3].

We found that three randomization centers violated an apriori rule regarding the restriction in the number of participants randomized between time strata. The study protocol allowed for exclusion of otherwise eligible participants based on this rule. Details of these excluded participants would be necessary to assess for differential allocation discretion [15].

We also found that in 3 strata there were baseline differences in the screening CT scan and all 4 baseline differences prognostically favored alteplase (Table 2). The study protocol allowed for investigators to exclude participants based on their subjective interpretation of the CT scan. According to the FDA PLA “when investigators observed what was subjectively felt to be significant early infarct signs on the screening CT scan, they would frequently proceed to re-question the patient and/or family as to the time of onset of the stroke” [3]. If this subjective criterion differed among enrolling investigators, such that time of symptom onset was re-questioned more frequently in one group than another, more favorable participants may have been enrolled in one group deterministically.

Stratification based on time of symptom onset was based on a pilot study that found treatment with alteplase within 90 min may be associated with early neurological improvement [16].

Therefore, imbalanced allocations in this time stratum that result in under-allocation of placebo compared to alteplase and vice versa in the later time stratum is compatible with potential selection bias. Four baseline differences were identified in the 91–180-min time stratum that prognostically favored alteplase; and none were found that prognostically favored placebo. These baseline differences explain the difference in the reported time stratum effect sizes (Table 3) for which a larger treatment effect is present in the 91–180-min stratum.

We reported an adjusted analysis of alteplase treatment effects based on the results of our sensitivity analyses. Although alteplase treatment effect size reductions were small, three out of seven previously positive reported results were revised as non-significant congruent with conclusions from our qualitative risk of selection bias assessment and sensitivity analysis. That the adjustments led to changes in some of the originally reported positive treatment effects and not others should be interpreted cautiously. Reasons independent of baseline imbalances such as differences in the inter-rater reliability of the endpoints and imputation of missing outcomes may explain this. For example, the inter-rater reliability of the mRS is lowest between a score of 1 and 2 which was the cutoff for a favorable and unfavorable outcome [17]. The trial dataset does not distinguish true values from imputed values for participants lost to follow-up. Therefore, we were unable to assess if the single imputation method used in the trial had differential effects on the endpoints; or if there were cases of participants who had some, but not all endpoints missing leading to a mixture of true values and imputed values for individual participants. Finally, in the case of the NIHSS endpoint in the 91–180-min stratum, it is logical that a baseline imbalance in the same score led to a larger reduction in treatment effect after adjustment of this imbalance than a different endpoint. These findings differ from acute ischemic stroke trials of mechanical thrombectomy for which there was high treatment consistency among similar endpoints used in the NINDS rt-PA Stroke Study [18].

A comparison to other thrombolytic and mechanical thrombectomy trials for acute ischemic stroke are worth considering when determining the risk of selection bias in the NINDS rt-PA Stroke Study. Thrombolytic treatment for acute ischemic stroke recommendations in the United States (US) and European Union (EU) are based on 7 RCTs [19]. Of these, the NINDS rt-PA Stroke Study and the ATLANTIS B RCT were the two largest RCTs performed in the US [20]. Both RCTs were sponsored by the same drug manufacturer, concomitantly enrolled participants, and had an identical study design with two major differences. The ATLANTIS B RCT enrolled participants from 3 to 5 h from symptom onset and did not stratify randomization by time from symptom onset [20]. Compared to the NINDS rt-PA Stroke Study, a similar proportion of alteplase allocated participants achieved favorable outcomes whereas the control arm performed better in ATLANTIS B. We believe selection bias in the NINDS rt-PA Stroke Study best explains why there is a large significant difference in the outcomes of placebo allocated participants relative to a small nonsignificant difference in alteplase allocated participants (Table 5). The two differences in trial design alone cannot meaningfully explain this discrepancy.

**Table 5:** Comparison of the NINDS rt-PA Stroke Study and the ATLANTIS B randomized controlled trials

Trial	Randomization Time Window After Stroke Symptom Onset	Randomization Strata	Cross Overs	Alteplase mRS 0-1	Placebo mRS 0-1
NINDS rt-PA Stroke Study	0-180 min	Time of Symptom Onset: 0-90 min and 91 to 180 min	21 placebo allocations that should have been alteplase.1 alteplase allocation that should have been placebo. (21:1)	45%^a^	25%^a^
		Clinical Center			
ATLANTIS B	180-300 min	Clinical Center	9 placebo allocations that should have been alteplase. 4 alteplase allocations that should have been placebo. (~ 2:1)	41.7%	40.5%
			**Risk Difference**	**3.5%, (−6.1 -13%),*p*** **= .48**	**15.4%, (6.8 - 23.9%),*p*** **< .001**

*Abbreviation*: *mRS* modified Rankin Scale

^a^Results reported from the 91–180-min time from symptom onset randomization stratum

Of the 7 thrombolytic RCTs, the NINDS rt-PA Stroke Study and the ECASS-3 RCT are the two individual RCTs with positive alteplase treatment effects while the others are neutral or negative [21]. Interestingly, the ECASS-3 RCT also suffered from baseline imbalances in covariates prognostically associated with outcomes [22]. This trial similarly used a stratified permuted block randomization scheme. A recently published revised analysis has convincingly shown that the study results are negative when appropriate covariate adjustments are made [22]. The results are similar to our adjusted analysis such that small changes in alteplase treatment effect sizes resulted in originally positive reported effects being revised as non-significant. The IST-3 RCT was unique compared to other thrombolytic trials in that a minimization algorithm was used for allocation [23]. Whether minimization or other randomization schemes such as minimal sufficient balance are better suited for acute stroke trials is an important area of future research [24]. Alternatively, Mandava and Kent have suggested that randomization alone is unlikely to achieve covariate balance in stroke trials and have used the NINDS rt-PA Stroke Study as an example [25, 26]. As with other publications citing the NINDS rt-PA Stroke Study, faulty randomization as an etiology of covariate imbalance was not considered. Covariate balance in large open label trials such as the IST-3 RCT and multiple mechanical thrombectomy trials do not strongly support their hypothesis [18, 23]. Mechanical thrombectomy acute stroke trials also likely benefitted from mandatory neuroimaging which is a potent predictor of stroke outcomes and superior baseline reporting. As noted by the authors, however, there is more compelling evidence of covariate imbalance after randomization in earlier phase stroke trials with smaller sample sizes.

Limitations of this risk of bias assessment are worth noting. Berger has popularized a statistical test to detect randomization subversion which was not performed and could support or deny the results of the reported assessment [9, 11, 12, 15, 27]. This test is most useful, however, in cases where randomization subversion is unobservable. In the current assessment, baseline differences in multiple prognostic factors in this trial exclusively favoring one group with corresponding imbalanced allocation seems most consistent with randomization subversion of the observable type. Additionally, this test requires knowledge of the actual allocation sequence which is not available. We were also unable to perform statistical testing to assess the possibility of over-stratification as the etiology of imbalanced allocations without knowledge of block sizes [28]. The use of hypothesis testing to compare baseline covariates in RCTs is questionable, but less so in cases where there is potential for selection bias [27]. Sensitivity analyses that evaluated baseline balance were only done in pre-specified strata and should not be confused with post-hoc subgroup analyses typically reported in RCTs [29]. Unlike post-hoc subgroup analyses, stratifying variables are identified a priori guarding against chance findings introduced by multiple hypothesis testing for subgroups identified a posteriori. Finally, the integrity of randomization within each stratum should be maintained unless faulty randomization has occurred. Baseline differences found between allocation groups were well established prognostic factors following stroke; were known to investigators prior to randomization; and in the case of baseline CT scan results could have been used to justify exclusion from randomization.

Our adjusted analysis shares similar limitations to all unplanned post-hoc reanalyses including those introduced by analytical methods not chosen apriori to dataset availability. We chose independent variables based on baseline imbalances found in randomization strata due to suspected randomization errors; and that had biologically plausible relationships with the dependent variables. The reported analysis differs from another frequently cited reanalysis in which independent variables were chosen based on an automated variable selection procedure [4]. The dependent variables were replicated from the original study. Although the original trial also reported a “global statistic” based on a generalized estimating equation, this is unlikely to change the reported results given the “global statistic” is a composite of the four individual endpoints. Additionally, the “global statistic” has no clinical meaning making the model results difficult to interpret and has lost favor as an endpoint in acute stroke trials [18].

As with all multivariable modeling, some subjective judgement is required. For example, in the 91–180-min stratum there was a greater proportion of patients that were receiving aspirin prior to treatment in the alteplase group. We assumed this favored the alteplase group as the relationship between aspirin treatment and more favorable outcomes in ischemic stroke is well described. If, however, a participant took aspirin just prior to randomization and was randomized to alteplase this may disproportionately increase the chance of intracerebral hemorrhage. Additionally, we used a *p* value threshold of .05 to determine a significant baseline imbalance within strata which is a common threshold and the threshold used for hypothesis testing by the trial authors; but this can be arbitrary for purposes of post-hoc analysis. For example, in the 91–180-min stratum there was an additional baseline imbalance in the any early CT finding variable favoring alteplase which was excluded from the model since hypothesis testing revealed a *p* value of .052. Other variables that were significantly different between the treatment groups, such as blood pressure, were removed due to inaccurate data collection as has been previously done [4]. These nuances are unlikely to be fully substantiated in post-hoc analyses. Although our adjusted analysis supports the presence of selection bias, it should not be considered a substitute for covariate balance and treatment effects produced by unbiased randomization. As such, the intent of the reported post-hoc analysis was not meant to determine the true alteplase treatment effects that would be determined by unbiased randomization. Rather, the reported revised treatment effects as well as the comparison to the ATLANTIS B RCT support that the treatment effects reported in the NINDS rt-PA Stroke Study are inflated due to probable selection bias. Limitations in post-hoc analyses have led some experts to claim that flawed randomization is “a serious bias uncorrectable by statistical analysis” [8]. Given the worldwide impact of thrombolytic therapy for acute stroke and lack of convincing replication, we believe there is equipoise to repeat an RCT similar to the NINDS rt-PA Stroke Study to determine the true alteplase treatment effect size.

Lastly, we have only judged randomization and the risk of selection bias; and no other potential sources of biases that could affect the reported treatment estimates including participant loss to follow-up. A total of 23 participants had endpoint values imputed [30]. Any potential bias associated with lost to follow-up may be difficult to gauge as the baseline characteristics of participants with missing outcomes and differential lost to follow-up between allocation groups was not reported in the original trial; or in trial documents and data reviewed for the current report. As previously noted, missing outcome data may be one possible explanation for differences in the revised alteplase treatment effects among endpoints. Additionally, imputed values were not distinguished from true values in the dataset making sensitivity analyses employing varying imputation methods unfeasible.

## Conclusion

This methodological review identified a high risk of selection bias in the NINDS rt-PA Stroke Study. Baseline imbalances in the trial were more likely due to randomization errors than random chance. An adjusted analysis revealed that the originally reported alteplase treatment effects were inflated, likely due to selection bias. An RCT similar to the NINDS rt-PA Stroke Study with unbiased randomization is likely required to determine the true alteplase treatment effect size. In the interim, treatment decisions and guideline recommendations based on the NINDS rt-PA Stroke Study should be done cautiously.

## Data Availability

All data generated or analyzed during this study are publicly available.

## Abbreviations

RCT: Randomized controlled trial
CT: Computed tomography
PLA: Product licensing application
ROB 2: Cochrane Risk of Bias 2
